# Dystrophinopathy presenting with arrhythmia in an asymptomatic 34-year-old man: a case report

**DOI:** 10.4076/1752-1947-3-8625

**Published:** 2009-07-24

**Authors:** Seth E Wakefield, Elliot L Dimberg, Steven A Moore, Brian S Tseng

**Affiliations:** 1 Department of Neurology, Massachusetts General Hospital and Harvard Medical School, Boston, MA 02114, USA; 2 Department of Neurology, Mayo Clinic, Jacksonville, FL 32224, USA; 3 Department of Pathology, Roy J and Lucille A Carver College of Medicine, University of Iowa, Iowa City, IA 52242, USA

## Abstract

**Introduction:**

Important clues in the recognition of individuals with dystrophin gene mutations are illuminated in this case report. In particular, this report seeks to broaden the perspective of early signs and symptoms of a potentially life-limiting genetic disorder. This group of disorders is generally considered to be a pediatric muscular dystrophy when in actual fact, this case report may represent a spectrum of subclinically affected adults.

**Case presentation:**

We present the diagnostic saga of a 34-year-old Caucasian man who had two liver biopsies for elevated liver enzymes and 16 years later presented with a cardiac arrhythmia amidst an emergent appendectomy which finally led to his specific genetic diagnosis.

**Conclusions:**

This genetic disorder can affect more than one organ, and in our patient affected both skeletal and cardiac muscle. Furthermore, liver function tests when elevated may erroneously implicate a liver disorder when they actually reflect cardiac and skeletal muscle origin. Presented here is a patient with Becker's muscular dystrophy and cardiomyopathy.

## Introduction

Dystrophinopathies are X-linked recessive disorders caused by loss of function mutations affecting the dystrophin gene [1]. The dystrophin gene consists of 79 exons which encode a huge 427 kDa membrane-associated protein found in some neurons and all muscle cells [2]. As large international genetic databases are maturing, it is becoming clear that dystrophinopathies present a spectrum of phenotypes and comorbidities. Becker's muscular dystrophy (BMD) and Duchenne's muscular dystrophy (DMD) are estimated to occur in 1:12,000 and 1:3500 male births, respectively. Intragenic deletions are the 3500 most common mutations leading to BMD or DMD. Patients with BMD exhibit milder progressive muscular dystrophy with ambulation maintained into the teenage and adult years [3]. On the other hand, patients with DMD tend to lose independent ambulation by 15 years of age and are life-limited by the end of the second decade usually due to cardiorespiratory compromise.

We present the case of a patient diagnosed with BMD in the middle of his fourth decade of life. Cardiac and skeletal muscle symptoms were absent until an emergent appendectomy when he was found to have a paradoxical arrhythmia. After a prolonged evaluation, our patient was shown to have a 2 bp out-of-frame deletion in exon 2 of his dystrophin gene, where the total gene has 79 exons.

## Case presentation

The patient had been a lifelong participant in sports, including baseball, soccer, basketball, cycling, volleyball, racquetball, running, and skiing, but noted occasional cramps while playing soccer that improved with rest. He could never do pull-ups nor run the mile in standard peer-group times. He was often told as a child and adult that he had large powerful-looking calves. He excelled academically, and attended graduate school in engineering. He currently runs his own internet business. The family history was non-contributory.

In late September 2005, this 34-year-old Caucasian man entered the emergency department with right lower quadrant pain confirmed to be acute appendicitis and he underwent an emergent appendectomy. During the operation, he developed a very irregular heartbeat. He was referred to a local cardiologist who diagnosed him with asymptomatic idiopathic dilated cardiomyopathy, NYHA class I to II. An electrocardiogram (EKG) showed his left ventricular ejection fraction to be 30%, where the normal range is 55-75%.

Prior medical and surgical history included a long history of elevated liver enzymes, having aspartate aminotransferase (AST) levels of 79-102 IU/L (normal 12-31 IU/L) and alanine aminotransferase (ALT) levels of 120-180 IU/L (normal 1-21 IU/L) which were incidentally found in screening tests before starting acne medication in 1990. A liver biopsy was performed at that time, and a second liver biopsy was performed five years later. Both biopsies found no pathologic hepatic abnormalities. In 1997, he presented with palpitations and after an EKG, was told that he had a left bundle branch block that was not investigated further.

An X-ray of the patient's chest in August 1997 showed cardiac size to be on the upper limits of normal. From October 2005 to December 2006, a local cardiologist repeated several studies including six EKGs, three echocardiograms, a nuclear treadmill stress test, two treadmill stress tests, and a cardiac catheterization, without any further diagnostic clarity for an underlying etiology. He was told that he likely had an idiopathic cardiomyopathy, possibly post-viral. He denied ever being ill to such an extent. His physical exam was thought to be normal otherwise.

In November 2006, he independently sought third and fourth opinions with cardiologists at two leading tertiary academic medical centers. At one center, he was tested and found to have an elevated serum creatine kinase (CPK) of 1011 IU/L (normal 0-250 IU/L). His physical exam showed mild symmetrical proximal muscle weakness and minimal weakness of the hamstrings, depressed stretch reflexes at his knees with downgoing toes and a significant calf pseudo-hypertrophy.

Echocardiography in November 2006 revealed severe left ventricle (LV) enlargement and an LV ejection fraction of 25-30% (normal = 55-75%). At that time, a blood DNA genetic test was ordered which revealed a presumed disease-causing mutation in his dystrophin gene. The DNA sequencing analysis revealed a 2 base pair deletion of guanidine and adenine in nucleotide positions 40 and 41 of exon 2 (total of 79 exons in the dystrophin gene). Dystrophin carrier testing in his mother was negative for this specific mutation, suggesting that a spontaneous mutation had occurred.

**Figure 1:**
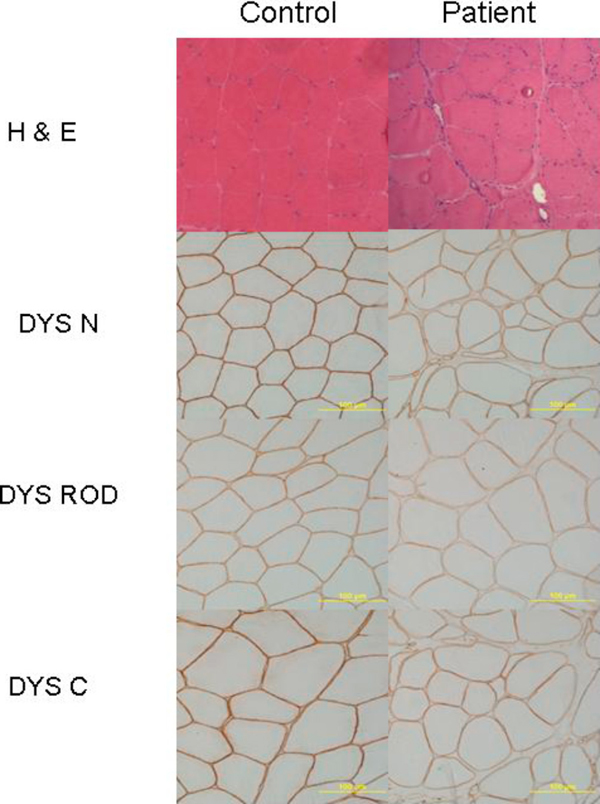
**Patient and control skeletal muscle biopsies: hematoxylin and eosin and anti-dystrophin immunoperoxidase staining is diffusely attenuated for three epitopes of dystrophin protein (N-terminus; rod domain; C-terminus, 20x)**.

**Figure 2:**
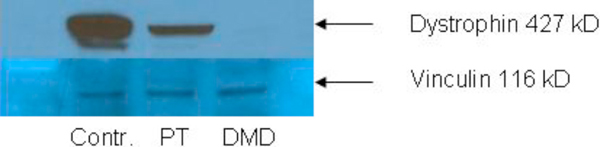
**Western blot of human skeletal muscle probed with 427 kDa monoclonal anti-dystrophin MANDRA 1 against the C-domain of the dystrophin protein (Sigma, St Louis, MO, USA) and reprobed with 116 kDa monoclonal anti-vinculin hVIN-1 (Sigma)**. Lane 1 is control skeletal muscle; Lane 2 is a biopsy sample from the patient of interest, at 33% of control levels of dystrophin protein [12]; Lane 3 is a biopsy sample from a patient with Duchenne's muscular dystrophy; 40 μg skeletal muscle per lane.

Skeletal and cardiac muscle biopsies were performed. A left vastus lateralis muscle (Figure 1) biopsy revealed chronic myopathy with fiber splitting, increased internal nuclei (up to 40%), and isolated necrotic and regenerating fibers. Immunohistochemical staining specific for three epitopes of dystrophin showed that the N-terminus was normal but the rod domain and C-terminus showed patchy staining unlike the control sections. Western blot of skeletal muscle showed that dystrophin was approximately normal size (≈427 kDa) and 33.7% of normal abundance compared to controls (Figure 2).

**Figure 3:**
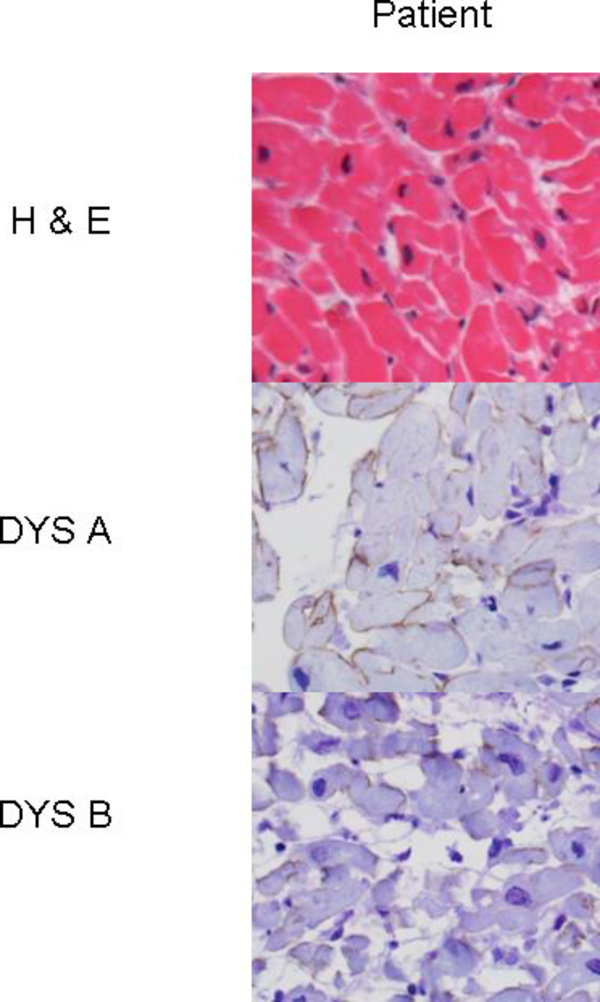
**Patient cardiac muscle biopsy: hematoxylin and eosin and anti-dystrophin immunoperoxidase staining at 40x**.

A cardiac muscle biopsy revealed no pathologic findings to support myocarditis, but mild fibrosis and increased variability in cardiac myocyte diameter and the nuclear morphology were consistent with a cardiomyopathy. Immunoperoxidase staining following antigen retrieval with proteinase K was performed on his wax-embedded cardiac biopsy. Dystrophin immunostaining with both the rod domain and amino-terminus antibodies using dysA and dysB, respectively, (Novocastra) were both markedly reduced (Figure 3) compared to control. Alpha-dystroglycan had a similar immunolabeling using the glycoepitope-specific antibody IIH6 (Upstate) supporting patient tissue integrity. No frozen cardiac tissue was available to perform a Western blot.

An implantable cardioverter defibrillator (ICD) was placed in July 2007 due to concerns of a paroxysmal life-threatening arrhythmia. At the time of writing, the patient is scheduled for an arrhythmia ablation procedure to remove an abnormal conduction pathway. He has been told that, in the future, if his heart problem continues to progress, he may need to be considered for cardiac transplantation.

## Discussion

This case report describes a healthy man who presented with a life-threatening arrhythmia on the operating table and was subsequently diagnosed with BMD, based on the clinical features, the genetic testing and the skeletal muscle analysis.

The patient had elevated liver enzymes 16 years before his arrhythmia. Liver disease was assumed so the patient underwent two liver biopsies which were both unrevealing. Skeletal and cardiac muscle disease can be found with elevations in soluble metabolic enzymes, for example, AST and ALT. Thus, blood test screening for both serum CPK and gamma glutamyl-transferase (GGT) should be considered to distinguish liver from muscle abnormalities.

Cardiac abnormalities are common in patients with BMD and may be the presenting symptom. In fact, cardiac complications are a more frequent cause of death in patients with BMD than in patients with DMD [4]. Angelini *et al*. found that 54% of electrocardiographic and 65% of echocardiographic data were abnormal for those patients with muscular dystrophy given extensive cardiac evaluation [5]. These patients experience LV dysfunction and could require a ventricular assist device or heart transplantation. Our patient required an ICD, and will have arrhythmia ablation surgery. Doing et al. reported that a trial of beta blockers and angiotensin converting enzyme inhibitors (ACEIs) can be a successful intervention, delaying or omitting the need for heart transplantation [6].

In 1996, Saito *et al*. showed that patients with BMD had poorer LV systolic function, as measured by pre-ejection time period/ejection time (PEP/ET); larger LV end diastolic dimension (LVDd), and a larger mitral valve annular size at end diastole compared to patients with DMD and with healthy controls [4]. They also showed that mitral regurgitation was present significantly more often in patients with BMD with cardiac failure compared to patients with BMD without cardiac failure.

Our patient showed a clinical picture consistent with BMD, despite a frame-shift mutation. As a further paradox, the frameshift DNA hypothesis predicts the DMD phenotype with out-of-frame mutations and the BMD phenotype is predicted for mutations that keep the reading frame intact. With this case in mind, diagnostic laboratories and physicians must be cautious not to offer overstated genetic results without validated population clinical data plus immunohistochemical data or a protein study.

Why does this specific dystrophin mutation in our patient affect his cardiac function more than his skeletal muscle which is mildly weak? In 1996, Angelini *et al*. conducted a large-scale survey of mild dystrophinopathies and found a sizable group of patients lacking severe muscle wasting but presenting with severe cardiac conditions [5]. They suggest that muscle up-regulates the expression of isoforms of dystrophin but the heart does not, an idea also presented by Milasin *et al*. [2].

In 2006, Aartsma-Rus *et al*. showed that only 2% of all patients have an out-of-frame deletion or duplication associated with a BMD phenotype [3]. In 2008, Kesari *et al*. published data showing 30% reading-frame exceptions in patients with DMD, especially with deletions at the 5' end of the gene [7]. In 1996, Angelini *et al*. showed that a deletion at the 5' end of the gene is usually associated with a rapid disease course; our patient contradicted this observation [5]. Mutations at the 5' end of the DMD gene can result in dystrophinopathy with substantial cardiac involvement [2]. Patients with dystrophin-deficient X-linked cardiomyopathy have dystrophin deletions or alternative splicing mutations that affect the 5' end of the gene, specifically the muscle promoter and muscle specific exon 1. In every case, the cardiac muscle was dystrophin-deficient while the skeletal muscle dystrophin level was normal. They also found that the heart muscle dystrophin transcripts in their patient with the 5' deletion were comparable to patients with XLDCM (X-linked dilated cardiomyopathy) [2].

## Conclusion

This patient was a diagnostic challenge for physicians including pediatricians, internists, family practitioners, neurologists, gastroenterologists and cardiologists. In 1997, Muntoni *et al*. reported two patients who presented with idiopathic dilated cardiomyopathy (IDCM) later proven to be caused by dystrophin gene deletions [8]. Myopathic symptoms may be delayed and mild in BMD, even when there is severe cardiomyopathy. Cardiac symptoms can appear in any individual with dystrophin gene mutations and can be progressive. Early identification may enable earlier prophylactic treatment with afterload reducing medications and possibly even corticosteroids which have been favorable in boys with DMD [9]. It took three cardiac specialists from leading academic cardiac hospitals to eventually clarify our patient's diagnosis from IDCM to cardiac dystrophinopathy with BMD.

It is clear that cardiac involvement should be anticipated in patients with BMD and will be progressive over time. In a large study of 68 patients with BMD by Nigro *et al*. in 1995, it was found that every patient over 30 years of age had cardiac involvement [10]. The rate of progression is unpredictable and a large percentage of patients will be affected with a disabling and potentially deadly dilated cardiomyopathy [11]. Mutations at the 5' end of the DMD gene pose a great threat combining mild skeletal muscle symptoms with severe cardiac involvement. Hopefully, this case report will help raise awareness of dystrophinopathy disorders when abnormalities of the liver screening tests, cardiomyopathy and mild skeletal muscle weakness are discovered.

## Patient's perspective

There are many others with subclinical BMD symptoms that go undiscovered until it is too late. Simple and inexpensive CPK blood work screening should be pursued in all infants/children, to find these conditions early. Persons with elevated liver enzymes, heart conditions such as cardiomyopathy, left bundle branch issues, and idiopathic causes should also be screened to avoid dangerous and unnecessary tests and procedures. Basic medication safety screening also needs to be increased, to protect patients and families from medications/substances that cause irreparable damage and con-artists who prey on their optimism and wallets. Avoiding detrimental effects and ineffective medications is the easiest, cheapest, and safest way to stabilize the quality-of-life of these patients. A clearinghouse of these data needs to be created so that supplements and medicines, which have been proven to be safe, effective, and helpful for patients with BMD with heart conditions, are easily known to all doctors and especially patients.

## Abbreviations

ACEI: angiotensin converting enzyme inhibitor; ALT: alanine aminotransferase; AST: aspartate aminotransferase; BMD: Becker's muscular dystrophy; CPK: creatine kinase; DMD: Duchenne's muscular dystrophy; EKG/ECG: electrocardiogram; GGT: gamma glutamyl-transferase; ICD: implantable cardioverter defibrillator; IDCM: idiopathic dilated cardiomyopathy; LV: left ventricle; LVDd: left ventricular end diastolic dimension; PEP/ET: pre-ejection time period/ejection time; XLDCM: X-linked dilated cardiomyopathy.

## Consent

Written informed consent was obtained from the patient for the publication of this case report and any accompanying images. A copy of the written consent is available for review by the Editor-in-Chief of this journal.

## Competing interests

The authors declare that they have no competing interests.

## Authors' contributions

SW drafted the manuscript, searched the literature, analyzed the data, and performed the Western Blot analysis. ED provided immunostudies of skeletal muscle and helped edit the manuscript. SM provided immunostudies of heart and helped edit the manuscript. BT examined the patient, searched the literature, obtained/analyzed the data and drafted the manuscript. All authors have read and approved the final manuscript.
